# Human gastric microbiota analysis of refractory *H. pylori* infection

**DOI:** 10.1038/s41598-024-66339-9

**Published:** 2024-07-07

**Authors:** Xianfeng Huang, Da-ya Zhang, Da Li, Yanting Lv, Shiju Chen, Feihu Bai

**Affiliations:** 1 Graduate School, Hainan Medical University, Haikou, 571199 China; 2 Department of Gastroenterology, The Second Affiliated Hospital of Hainan Medical University, Yehai Avenue, #368, Longhua District, Haikou, 570216 Hainan Province China; 3 The Gastroenterology Clinical Medical Center of Hainan Province, Haikou, 570216 China

**Keywords:** Refractory *H. pylori* infection, Gastric microbiota, 5R16S rRNA sequencing, Bacteria functional prediction, Gastroenterology, Medical research, Pathogenesis

## Abstract

*H. pylori* infection is gaining increasing attention, but detailed investigations into its impact on gastric microbiota remain limited. We collected gastric mucosa samples from 47 individuals divided into three groups: 1. Group HP: patients with initial positive *H. pylori* infection (25 cases); 2. Group ck: *H. pylori*-negative patients (14 cases); 3. Group DiffHP: patients with refractory *H. pylori* infection (8 cases). The samples were analyzed using 16S rDNA sequencing and functional prediction with PICRUSt. Group HP showed differences in flora distribution and function compared to Group ck, while Group DiffHP overlapped with Group HP. The abundances of *Aeromonas piscicola*, *Shewanella algae*, *Vibrio plantisponsor*, *Aeromonas caviae*, *Serratia marcescens*, *Vibrio parahaemolyticus*, *Microbacterium lacticum*, and *Prevotella nigrescens* were significantly reduced in both Group DiffHP and Group HP compared to Group ck. *Vibrio shilonii* was reduced only in Group DiffHP compared to Group ck, while *Clostridium perfringens* and *Paracoccus marinus* were increased only in Group DiffHP. LEfSe analysis revealed that *Clostridium perfringens* and *Paracoccus marinus* were enriched, whereas *Vibrio shilonii* was reduced in Group DiffHP compared to Group ck at the species level. In individuals with refractory *H. pylori* infection, the gastric microbiota exhibited enrichment in various human diseases, organic systems, and metabolic pathways (amino acid metabolism, carbohydrate metabolism, transcription, replication and repair, cell cycle pathways, and apoptosis). Patients with multiple failed *H. pylori* eradication exhibited significant changes in the gastric microbiota. An increase in *Clostridium perfringens* and *Paracoccus marinus* and a decrease in *Vibrio shilonii* appears to be characteristic of refractory *H. pylori* infection.

## Introduction

The gastric mucosal environment was traditionally considered sterile, but the discovery of *H. pylori* challenged this traditional view^1^. *H. pylori* has been linked to various gastric disorders, such as chronic gastritis, peptic ulcer disease, gastric mucosa-associated lymphoid tissue (MALT) lymphoma, and gastric cancer. The World Health Organization (WHO) categorizes *H. pylori* as a Class I carcinogen^2,3^. Additionally, *H. pylori* is linked to several extragastric diseases such as iron deficiency anemia, vitamin B12 deficiency, type 1 diabetes mellitus, neurological disorders, and cardiovascular diseases^4^. However, due to the widespread use of antibiotics and increasing antibiotic resistance, eradication treatment failures are becoming more common, leading to the emergence of refractory *H. pylori* infections. Consequently, refractory *H. pylori* is gaining increased attention.

The unique gastric microenvironment, characterized by low pH, mucus, and peristalsis, makes the stomach a complex organ and inhibits the growth of most bacteria. As a result, the gastric microbial composition differs significantly from that in other body regions^5^. Versalovic et al. identified diverse gastric microbiota using 16S rRNA sequencing^6^. The human stomach hosts a complex microbiota primarily composed of the *Proteobacteria, Firmicutes, Actinobacteria* and *Fusobacteria species*^7^, which are the most abundant and cultured phyla. Complex interactions between *H. pylori* and other microbial communities within this unique gastric microecological setting are associated with *H. pylori* infection in humans^8^. Recent research has concentrated on the role of changes in gastric microbiota in the development of gastric diseases and their relationship with *H. pylori* infection^7,9–12^. However, the dynamics of gastric microbiota in refractory *H. pylori* infections remain underexplored, and the interaction patterns remain poorly understood.

This study employed 16S rRNA amplicon sequencing to analyze the gastric flora of patients with primary *H. pylori* infection, non-*H. pylori* infection, and refractory *H. pylori* infection. The objective was to elucidate the characteristics of the gastric flora associated with refractory *H. pylori* infection and to offer a novel theoretical foundation for future treatment approaches.

## Methodology

### Patients and samples

Gastric sinus mucosa samples for this study were collected from patients undergoing gastroduodenoscopy and 13C/14C-urea breath test at Qionghai People's Hospital, Sanya Central Hospital, Hainan Second People's Hospital, Dongfang People's Hospital, and the Second Affiliated Hospital of Hainan Medical University in Hainan Province between August and October 2023. The inclusion criteria were: (1) aged 18 to 80. (2) Patients with a BMI less than 23 kg/m^2^. (3) No antibiotics, bismuth, proton pump inhibitors (PPIs), or H2 receptor antagonists have been used in the past six months. (4) Presence of symptoms such as abdominal pain, bloating, acid reflux, belching, nausea, vomiting, heartburn, chest pain, or black stool. (5) Voluntary participation and signed informed consent. Antrum biopsy samples were obtained for microbiota analysis. The patients were categorized into three groups based on *H. pylori* status and therapy: Group Hp: treatment patients with *H. pylori* infection; Group ck: *H. pylori*-negative patients; and Group DiffHP: patients with refractory *H. pylori* infection. The study was approved by the Ethics Committee of the Second Affiliated Hospital of Hainan Medical University (2023-KCSN-Y13), and informed consent was secured from all participants prior to endoscopy.

### Relevant definitions

Refractory *H. pylori* infection is characterized by a persistently positive result on non-serological *H. pylori* tests (such as breath, fecal, or gastroscopic tests) at least four weeks after completing two or more rounds of first-line *H. pylori* eradication therapy, as recommended by current guidelines, and after discontinuing any medication that might affect test sensitivity, such as PPIs^11^.

The gastric sinus mucosa is located at the lowest point of the stomach, with the upper part connected to the gastric body and the lower part to the pylorus and duodenum. It lies between the plane of the gastric angle incision and the pylorus. *Helicobacter pylori* can survive in the stomach's highly acidic environment and firmly attach to the gastric mucosal surface, especially the antral mucosa, through strong motility, adhesion, and urease production, leading to disease development. Furthermore, the inflammatory response to *H. pylori* is more intense in the gastric sinus than in the gastric body. Therefore, gastric sinus mucosa was chosen for biopsy samples.

### Bacterial genomic DNA extraction

Samples were collected, processed, and sequenced as described in a previous study^13^. Briefly, frozen tissue samples (40–70 mg) were extracted using the CTAB method. All negative controls were processed following the same protocols. To mitigate the risk of contamination from hospital and laboratory environments and the diverse stages of sample handling and processing, a range of controls were implemented. These controls encompassed sampling controls, DNA extraction controls, and no-template PCR amplification controls.

### Amplification and sequencing of 5R 16S rRNA

Unlike microbial samples with high biomass (e.g., feces), microbial biomass in gastric mucosal tissue is extremely low and heavily influenced by host DNA. Amplification of intratumoral bacteria is often less successful when using conventional 16S regions (e.g., V3V4). 5R 16S sequencing was used for this project, involving multiplex PCR amplification and sequencing of five regions on the 16S rRNA gene. The sequencing of libraries was conducted on the Illumina NovaSeq 6000 system. Subsequently, reads were demultiplexed for each sample, filtered, and aligned to the five amplified regions based on the primers' sequences. Utilizing the Short MUltiple Regions Framework (SMURF) method, read counts from these regions were integrated to generate a coherent profiling result through the resolution of a maximum likelihood problem^14^. The reference database employed was the GreenGenes database (the May 2013 version, with some improvements). As described in a previous study^13^, filters were utilized to identify and eliminate contamination. Sequence reads were normalized from each sample to mitigate the influence of low-abundance noise on subsequent analyses. Furthermore, bacteria in negative control samples were used to identify and remove contaminants from the sampling and experimental processes.

### Bioinformatics analysis

The raw data underwent analysis in QIIME software (version 2) using bioinformatics techniques with default parameters. Sequences showing a similarity of ≥ 97% were grouped into operational taxonomic units (OTUs). To minimize the impact of low-abundance noise on downstream analyses, the sequence reads for each sample were normalized by filtering out samples with a total read count of < 1000 (including negative controls) and bacterial data with a relative abundance of < 10–4. Then, contaminant bacteria at both sampling and experimental stages were identified based on the prevalence of bacteria in negative control samples. A threshold of 50% prevalence was set in this study to determine contaminating bacteria. Alpha diversity was assessed (Chao1, Shannon), while beta diversity was evaluated through weighted UniFrac principal coordinate analysis (PCoA). Differences in bacterial composition between groups were examined using the Metastats algorithm. Furthermore, the Linear Discriminant Analysis (LDA) effect size (LEfSe) algorithm was applied to detect biomarkers displaying discrepant abundance across the different groups.

### Metagenomics by PICRUSt

The functions of different macrogenomes across various sample types were predicted using PICRUSt. Additionally, the software calculated the relative abundances of the Kyoto Encyclopedia of Genes and Genomes (KEGG) pathways. Functional differences between groups were analyzed using the Tukey HSD test in the R Vegan package (version 2.5.3).

### Statistical analysis

Statistical analysis was performed utilizing SPSS 25. Continuous variables were expressed as mean ± standard deviation (SD), and comparisons between the two groups were made utilizing the T-test or Wilcoxon rank sum test. Categorical variables were expressed as frequencies and percentages [n (%)], and group comparison was conducted using chi-square or trend chi-square tests. One-way ANOVA was utilized to compare the three patient groups. P values were corrected utilizing FDR analysis of KEGG pathways. All statistical tests were conducted as two-tailed tests. Results with P-values > 0.05 are considered non-significant, P-values ≤ 0.05 are marked as *, and P-values ≤ 0.01 are marked as **.

### Ethics approval and consent to participate

The protocol was approved by the Clinical Ethics Committee of the Second Affiliated Hospital of Hainan Medical University and performed per Helsinki's Declaration (approval number: 2023-KCSN-Y13). All participants provided written informed consent for data collection and storage.

## Results

### Patient characteristics

A total of 47 patients were enrolled in this study, with 47 samples collected. Group HP included 25 patients, Group ck included 14 patients, and Group DiffHP included 8 patients. No significant variations in age, gender, or BMI were observed across the three groups. The clinical information of the patients is summarized in Table 1.

**Table 1:** Clinical baseline data among three groups.

Group	Ck (14)	HP (25)	DiffHP(8)	P value
Age (year)	48 (28–63)	54 (44–64)	58 (52–62)	0.399
Sex (female, %)	9 (64.3)	13 (52)	4 (50)	0.725
BMI (Kg/m^2^)	21.7 (17.1–30.0)	24 (15.6–31.9)	22.9 (18–28)	0.791

### Rarefaction curves

**Figure 1:**
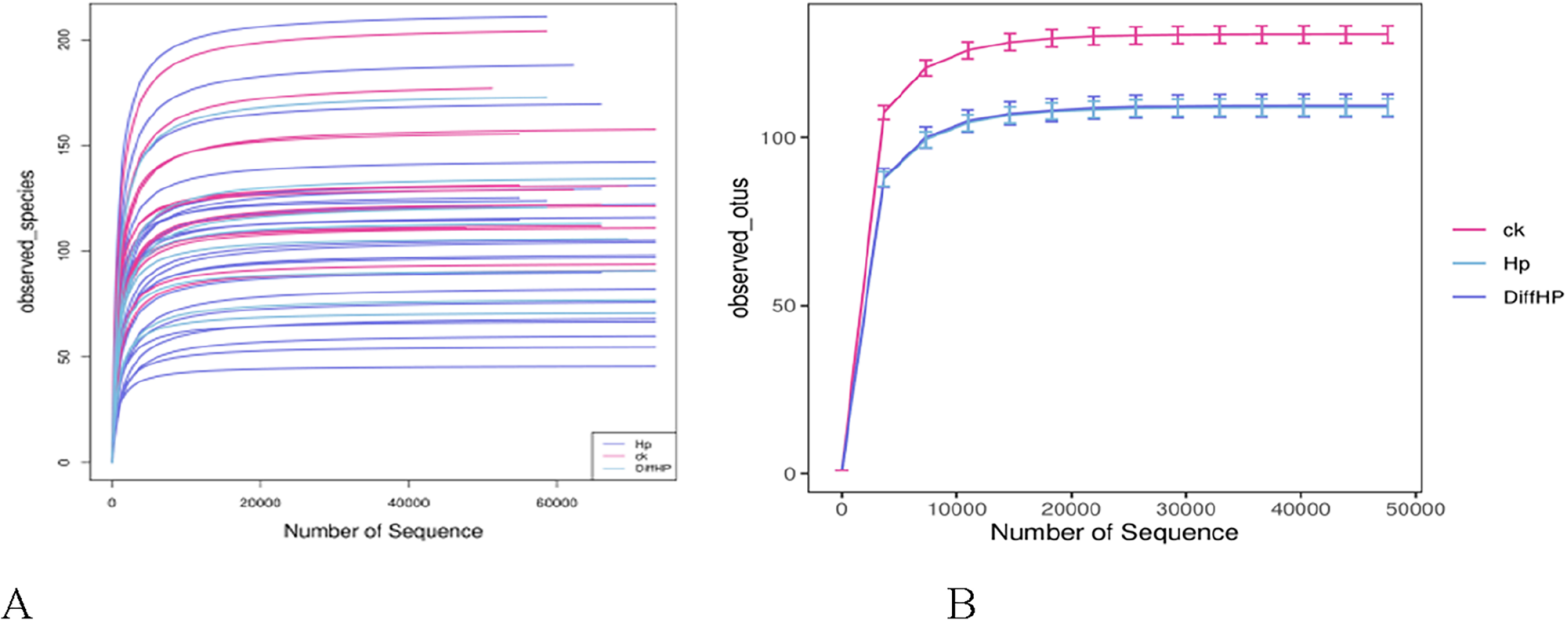
Rarefaction curves based on independent samples (**A**) and groups (**B**). CK: *H. pylori*-negative patients; HP: treatment-naïve patients with *H. pylori* infection; DiffHP: patients with refractory *H. pylori* infection.

The rarefaction curves for each sample gradually reached a plateau as sequencing depth increased (Fig. 1A), suggesting that the sequencing data were sufficient. As displayed in Fig. 1B, the observed species followed the order of abundance as Group ck > Group Diff HP > Group HP.

### Alpha diversity

**Figure 2:**
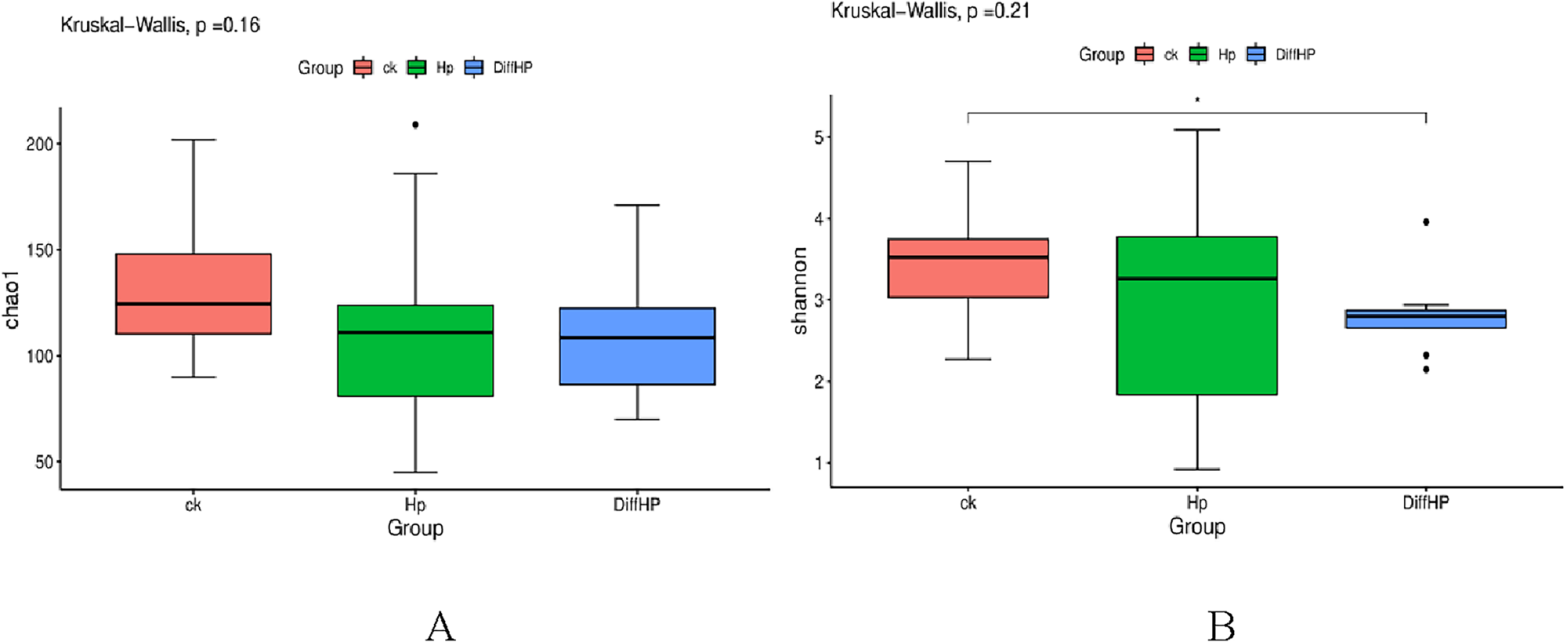
Comparisons of bacterial diversity index among groups. Chao1 index (**A**), Shannon index (**B**). CK: *H. pylori*-negative patients; HP: treatment-naïve patients with *H. pylori* infection; DiffHP: patients with refractory *H. pylori* infection.

Figure 2 illustrates the Alpha diversity ranking of the groups as follows: Group ck > Group HP > Group Diff Hp. However, no significant difference in Alpha diversity was observed among the three groups (*P* > 0.05).

### Beta diversity

**Figure 3:**
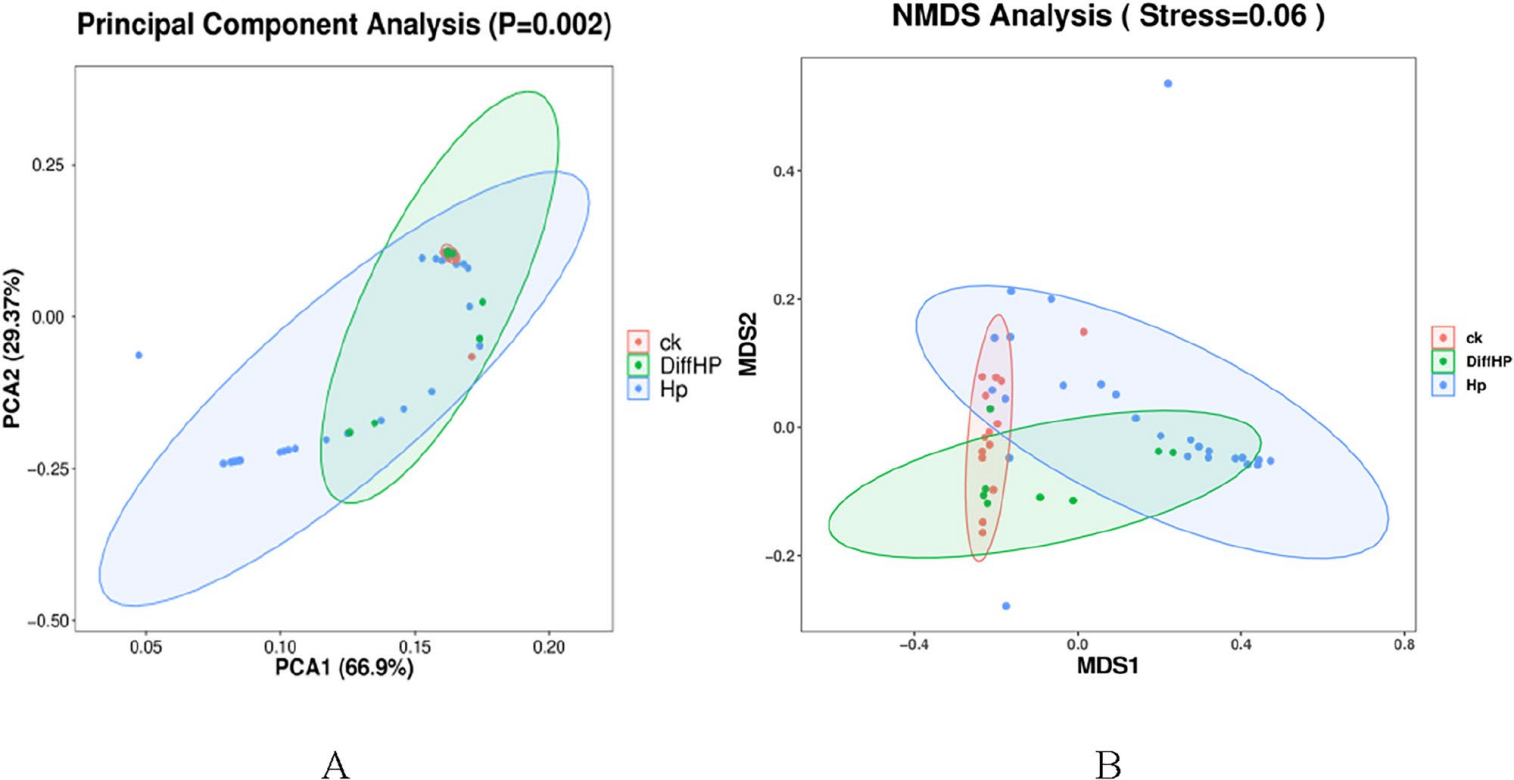
Comparisons of Beta diversity among groups. PCoA based on weighted UniFrac distance of the gastric microbiota among groups (**A**), NMDS analysis (**B**). CK: *H. pylori*-negative patients; HP: treatment-naïve patients with *H. pylori* infection; Diff HP: patients with refractory *H. pylori* infection.

A significant difference in beta diversity was observed among the three groups (*P* < 0.05) (Fig. 3A). Additionally, the distances indicated a high similarity between Group HP and Group DiffHP. The results showed a stress value of 0.06, suggesting that the graph has meaningful explanatory significance (Fig. 3B).

### Species composition and relative abundance

**Figure 4:**
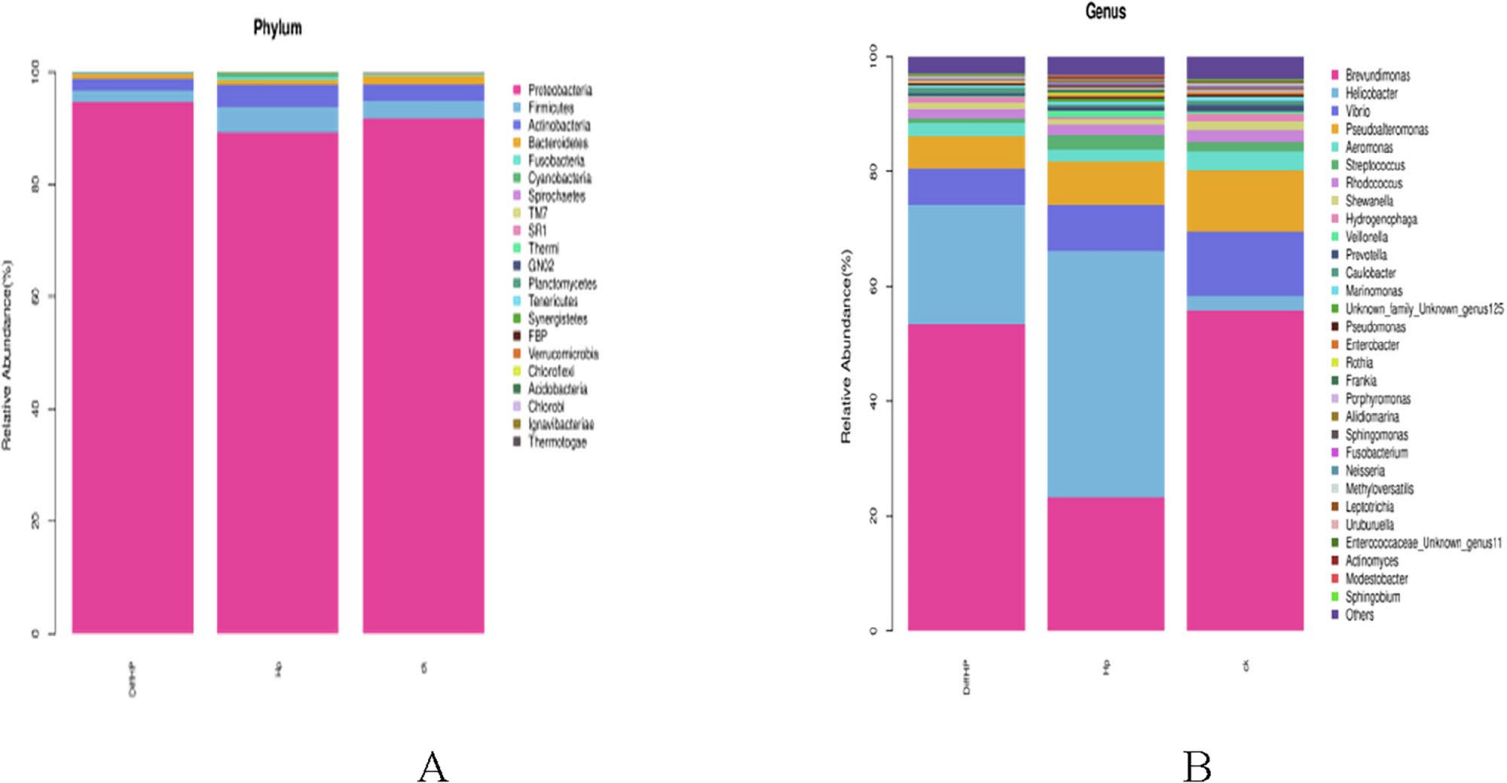
Relative abundance of gastric microbiota species (**A**) level and genera (**B**) level among groups. CK: H. pylori-negative patients; HP: treatment-naïve patients with *H. pylori* infection; DiffHP: patients with refractory *H. pylori* infection.

The predominant species observed in the three sample groups were *Firmicutes, Bacteroidetes, Actinobacteria*, and *Proteobacteria.* At the phylum level (Fig. 4A), the relative abundance of *Proteobacteria* was higher in Group DiffHP (94.67%) compared to Group HP (89.33%) and Group CK (91.77%), while the content of *Firmicutes* (2.03%), *Actinobacteria* (2.18%), and *Bacteroidetes* (0.75%) was lower than in the other groups. At the genus level (Fig. 4B), *Vibrio and Pseudoalteromonas* were most abundant in Group CK, followed by Group HP and then Group DiffHP. The relative abundance of *Brevundimonas* and *Aeromonas* was highest in Group CK, followed by Group DiffHP and Group HP. In contrast, *Pseudomonas* was most abundant in Group DiffHP (0.41%), followed by Group CK (0.32%) and Group HP (0.28%). It was the only genus-level bacterium that became dominant in Group DiffHP, surpassing the other two groups. The relative abundance of *Helicobacter* was significantly higher in Group HP and Group DiffHP. Analyzing the relative abundance of *Helicobacter*, Group DiffHP (20.69%) had a lower abundance than Group HP (42.87%).

**Figure 5:**
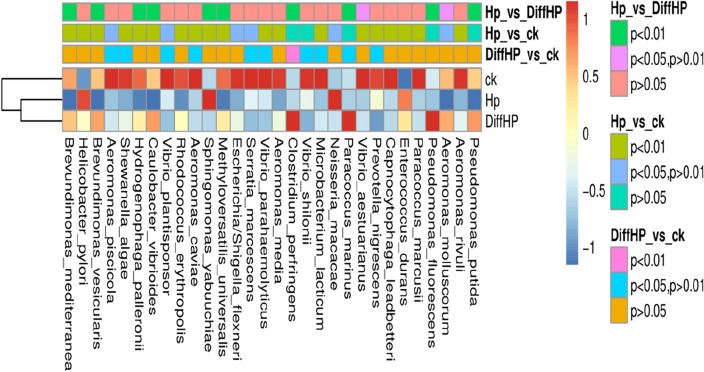
Heatmap of different species among three groups. CK: *H. pylori-negative* patients; HP: treatment-naïve patients with *H. pylori* infection; DiffHP: patients with refractory *H. pylori* infection.

**Figure 6:**
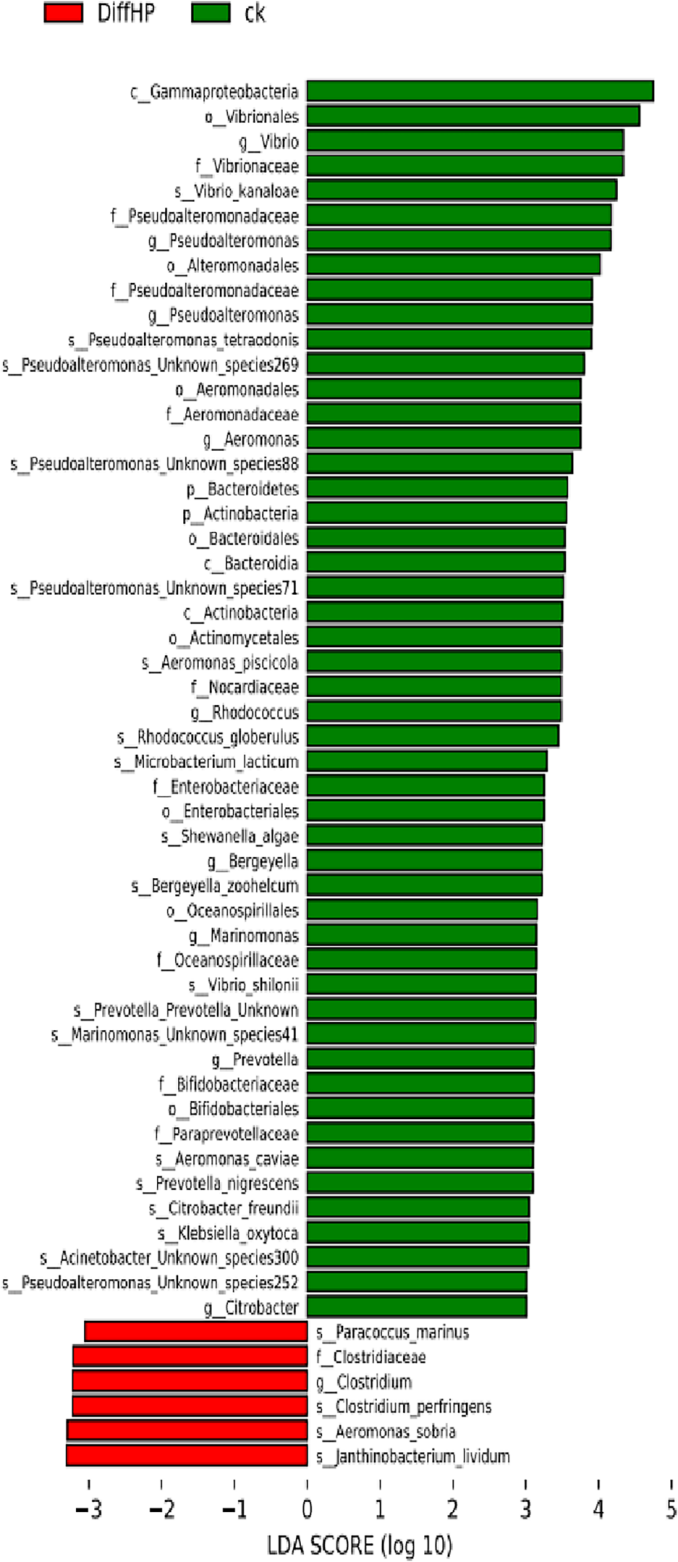
Identification of differential bacteria between Group ck and the Group DiffHP by LEfSe analysis. CK: *H. pylori*-negative patients; DiffHP: patients with refractory *H. pylori* infection.

The beneficial bacterium *Lactobacillales* was found to be enriched in Group HP (2.93%) compared to Group DiffHP (1.14%) at the order level (Fig. S1). Differential analysis of the gut microbiome (Fig. 5) revealed that the abundances of individual species, including *Aeromonas_piscicola, Shewanella_algae, Vibrio_plantisponsor, Aeromonas_caviae, Serratia_marcescens, Vibrio_parahaemolyticus, Microbacterium_lacticum,* and *Prevotella_nigrescens* were significantly reduced in the gut microbiome of both Group DiffHP and Group HP compared to Group CK. The abundance of individual species in *Vibrio_shilonii* was reduced only in Group DiffHP compared to Group CK. In contrast, the abundance of species in Clostridium_perfringens and Paracoccus_marinus increased only in Group DiffHP compared to Group CK. Key bacterial members serving as biomarkers among the three groups were identified utilizing LEfSe. The LDA scores in Group DiffHP compared with Group ck showed that *Firmicutes (f__Clostridiaceae, g_Closteidium), Proteobacteria (s_Aeromonas_sobria, s_Janthinobacterium_lividum, s_Paracoccus_marinus)* were enriched (Fig. 6). *Proteobacteria (o__Campylobacterales, c__Epsilonproteobacteria, f__Helicobacteraceae, s_Helicobacter_pylori)* were enriched in Group HP compared to Group CK (Fig. S2A). *g__Prevotella, f__Paraprevotellaceae, s__Sphingomonas_yabuuchiae and g__Sphingomonas* were enriched in DiffHP compared with group HP (Fig. S2B).

### Metagenomic function prediction by PICRUSt

**Figure 7:**
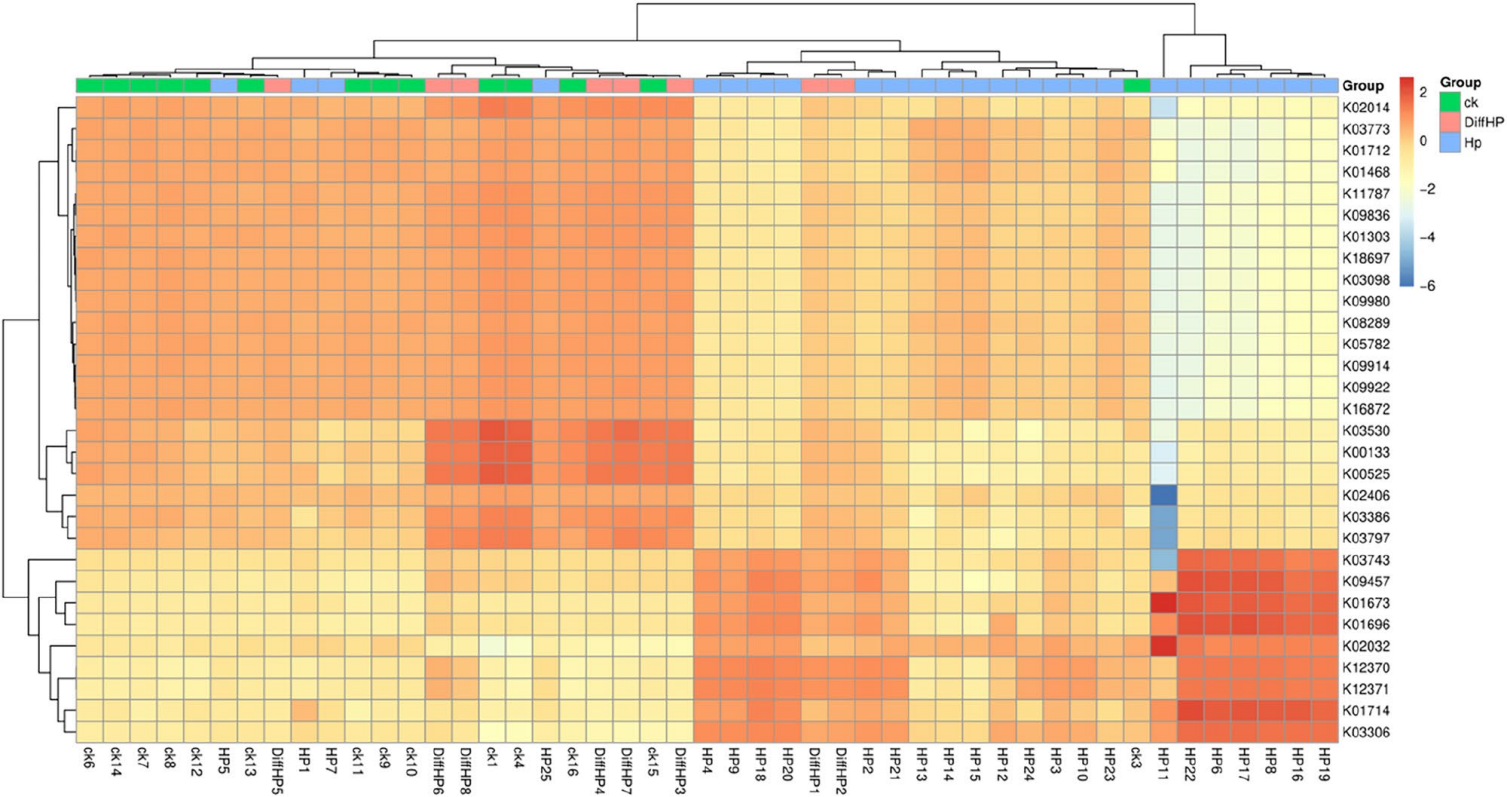
KEGG ontologies heatmap.

**Figure 8:**
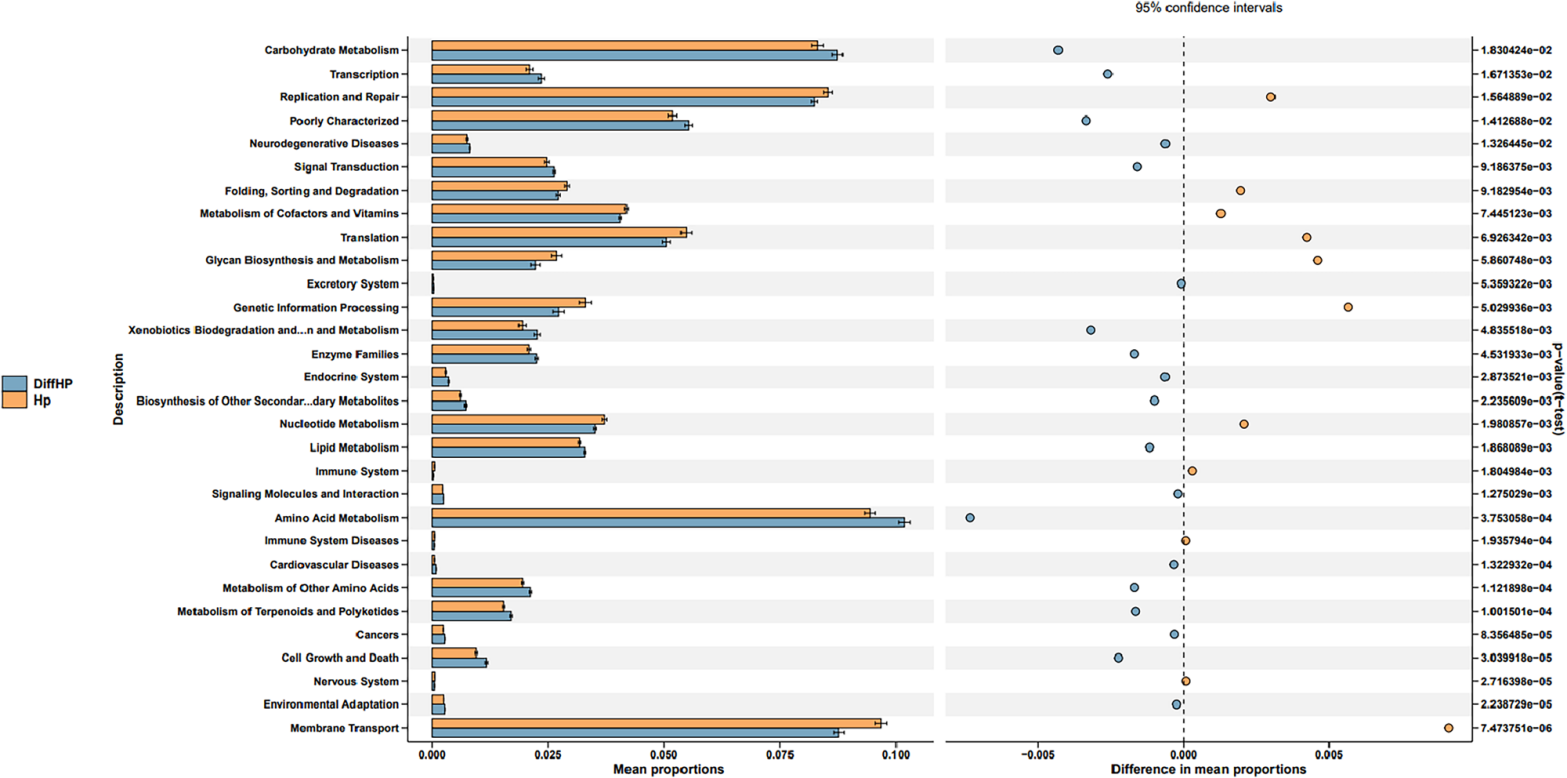
The predicted gastric microbiota function in the KEGG pathway at levels 2 between group DiffHP and group. HP: treatment-naïve patients with *H. pylori* infection; DiffHP: patients with refractory *H. pylori* infection.

Figure 7 illustrates the heatmap showing the distribution of KEGG ontologies across the three groups. The clustering analysis revealed distinct patterns of KEGG ontology blocks between Group CK and Group HP, suggesting differences in bacterial gene functions. An overlap between Group DiffHP and Group HP was observed, prompting further analysis of KEGG orthology (KO) differences between these two groups. At level 2 pathways, Group DiffHP exhibited a significantly higher abundance of amino acid and carbohydrate metabolism than Group HP. Transcription, replication and repair, and various types of metabolism were also more abundant in Group DiffHP. In contrast, genetic information processing was dominated by Group HP (Fig. 8). In level 1 pathways, Group DiffHP showed higher abundances of human diseases, organismal systems, and metabolic pathways compared to Group HP (Fig. S3A). At level 3, the abundance of most metabolic pathways varied between the groups. Group DiffHP had a higher abundance of membrane and intracellular structural molecules, which are important components for maintaining cellular homeostasis. Group HP had a higher abundance of amino sugar and nucleotide sugar metabolism pathways involved in cell growth, differentiation, and metabolism. These findings suggest that patients with *H.pylori* infection have vigorous cell metabolism (Fig. S3B).

## Discussion

The human gastrointestinal microbiota is often considered a functional organ that maintains a close, mutually beneficial symbiotic relationship with its host. It plays a vital physiological role in health, including supporting the host immune system and synthesizing essential nutrients^15^. Approximately 58% of the global population is colonized with *H. pylori*^16^. Previous investigations have shown that *H. pylori* infection significantly impacts the gastrointestinal mucosal microbiota^7,9–12^. Eun et al.^17^ proposed significant variations in the composition and richness of the gastric microbiota in individuals with gastric cancer, intestinal metaplasia, and chronic gastritis, particularly in those dominated by *H. pylori*. Furthermore, the oncogenic effect of *H. pylori* may partly result from its impact on the gastric commensal flora, creating a favorable environment for gastric cancer development^7^.

The updated Sixth National Consensus on the Management of H. pylori Infections 2022 defined refractory *H. pylori* infection as unsuccessful eradication treatment with ≥ 2 consecutive standardized regimens of different drug combinations, affecting at least 5% to 10% of patients^18^. Refractory *H. pylori* is increasingly gaining attention, and drug resistance analyses are being conducted in various regions to provide individualized treatment for patients. However, research into the gastric microbiota of patients with refractory *H. pylori* infection is sparse, and the interaction patterns within this group remain poorly understood. This study aims to deepen our understanding of the gastric microbiota in refractory *H. pylori* infections by observing their interaction patterns (positive and negative). Identifying microbial genera that can restore gastric microbiota harmony may offer alternative strategies to counter gastric ecological dysregulation caused by *H. pylori* and other pathogenic bacterial genera^19^.

In patients infected with *H. pylori*, the predominant bacterial classes in the stomach were Firmicutes, Proteobacteria, and Actinobacteria, indicating that *H. pylori* induces gastric bacterial dysbiosis^12,20,21^ Our study found that in the refractory *H. pylori* group, the relative abundance of *Pseudomonas* increased, surpassing other groups to become the dominant genus. A histogram showed that the refractory *H. pylori* group had a higher relative abundance of *Proteobacteria* than the first-time positive *H. pylori* group, while the abundances of *Firmicutes* and *Actinobacteria* were lower. However, LEfSe analysis indicated no statistical significance. The results could be attributed to medication^22–24^ or subtle alterations in the gastric microbiota induced by refractory *H. pylori*. The remaining *H. pylori* may have developed antibiotic resistance through mutations in drug-resistant genes, making eradication more difficult. Additionally, *H. pylori* eradication leads to microbiota disruption, potentially explaining why *Clostridium perfringens*, identified in this study, increased in the refractory *H. pylori* group and became the dominant flora, surpassing *H. pylori* in abundance. Consequently, the refractory *H. pylori* group exhibited lower abundance than the group testing positive for *H. pylori* for the first time. Previous studies^20,25^ have suggested that the ratio of *Bacteroidetes* could serve as a potential indicator of gastric mucosal inflammation, and an elevated *Firmicutes*/*Bacteroidetes* ratio might indicate an increased risk of tumorigenesis. However, our study did not assess the patients' gastric conditions, including the presence or severity of inflammation or other gastric disorders, preventing us from offering additional insights.

Compared to *H.pylori*-negative population, LDA scores using LEfSe showed that an increase in *Clostridium_perfringens*, *Paracoccus_marinus*, and a decrease in *Vibrio_shilonii* appear to characterize refractory *H. pylori* patients. *Clostridium perfringens* is an anaerobic, Gram-positive, spore-forming bacillus known to cause acute gastrointestinal infections in humans. These infections can vary from mild diarrhea to severe conditions such as necrotizing enterocolitis and myonecrosis^26^. Further study of *Clostridium_perfringens* provides a novel insight into the pathogenesis of Refractory *H. pylori* and offers novel treatment strategies. Compared to refractory *H. pylori* patients, the initial positive group showed increased *Prevotella* and *Sphingomonas*. *Prevotella* colonization can lead to changes in microbial metabolism, exacerbating intestinal inflammation and potentially triggering systemic autoimmune reactions. *Sphingomonas* has a cell membrane containing sphingolipids that are more hydrophobic than lipopolysaccharides and possess effective metabolic and genetic regulation mechanisms. This genus has significant potential for synthesizing velan gum, environmental remediation, and promoting plant growth.

Previous studies^19,27,28^ mentioned that the microbial diversity of *H. pylori* may correlate with different stages of gastric progression and physiological changes. In the early stages of chronic *H. pylori* infection, the bacterium gains a survival advantage in acidic conditions, becoming the dominant species in the stomach and resulting in reduced biodiversity of the gastric microbiota. However, prolonged *H. pylori* infection can lead to gastric atrophy, raising gastric pH. This elevation creates an environment unsuitable for acid-dependent bacteria and ultimately suppresses *H. pylori* growth through nutrient competition or other unidentified mechanisms^29,30^. However, studies on *H. pylori* infection, whether in adults^31^ or children^32^, primarily focus on comparing the gastric microbiota of infected and uninfected groups. The findings consistently showed lower bacterial diversity in *H. pylori*-positive gastric specimens than in negative ones. Recent consensus on *H. pylori* has confirmed that antibiotics significantly affect the gut flora, and their use in eradication therapy can promote the development of resistant strains and alter the diversity of species within the gut microbiota^33^. Regarding beneficial stomach flora, a higher relative abundance of *Lactobacillus* was observed in individuals with first-time *H. pylori* infections compared to those with refractory infections. Probiotic supplementation therapy, currently recognized for its potential^34^, enhances antibiotic efficacy and mitigates antibiotic-induced alternations in the composition of intestinal flora^35^.

The abundance of cell cycle and apoptosis pathways is significantly greater in the *H. pylori* positive group compared to the negative group, suggesting a potential link to the development of diseases such as colorectal cancer and small cell lung cancer. Future research will focus on confirming the association between *H. pylori* and these diseases. Previous epidemiological research has associated *H. pylori* with a variety of extragastric diseases^36,37^, including neurological, cutaneous, hematological, ophthalmic, cardiovascular, metabolic and allergic diseases, vitamin B12 deficiency^38^ and iron-deficiency anemia^39^. Infectious diseases upregulated pathways that align with *H. pylori*'s clinical pathogenicity, which has been linked to gastric cancer^21^. The small sample size may bias the results, necessitating more clinical data and additional studies using animal models for future validation.

Our study has several limitations. First, the small sample size may not comprehensively analyze changes in the gastric microbiota associated with refractory *H. pylori* infection. Second, our study did not control dietary habits, which can influence gut microbiota, potentially introducing bias. Third, we did not conduct animal experiments to explore the potential pathogenic mechanisms of refractory *H. pylori* infection. In future work, we plan to enhance our research by using a mouse model to investigate these mechanisms and to analyze the survival strategies of the key bacterium identified in this study, *Clostridium perfringens*.

## Conclusion

In summary, *H. pylori* infection substantially affects the diversity, composition, and function of the gastric microbiota. Patients with multiple unsuccessful attempts at *H. pylori* eradication exhibit significant alterations in their gastric microbiota. Notably, an increase in *Clostridium perfringens* and *Paracoccus marinus* and a decrease in *Vibrio shilonii* characterizes the gastric environment of patients with refractory *H. pylori* infection.

### Supplementary Information

Supplementary Figures.

## Data Availability

The datasets generated and/or analyzed during the current study are available from the corresponding author upon reasonable request.

## Abbreviations

*H. pylori*: Helicobacter pylori
PPI: Proton pump inhibitor
UBT: Urea breath test
PCoA: Principle coordinate analysis
LDA: Linear Discriminant Analysis
KEGG: Kyoto Encyclopedia of Genes and Genomes
SD: Standard deviation
