# Publisher Correction: Human gastric microbiota analysis of refractory *H. pylori* infection

**DOI:** 10.1038/s41598-024-68540-2

**Published:** 2024-07-31

**Authors:** Xianfeng Huang, Da-ya Zhang, Da Li, Yanting Lv, Shiju Chen, Feihu Bai

**Affiliations:** 1 Graduate School, Hainan Medical University, Haikou, 571199 China; 2 Department of Gastroenterology, The Second Afliated Hospital of Hainan Medical University, Yehai Avenue, #368, Longhua District, Haikou, 570216 Hainan Province China; 3 The Gastroenterology Clinical Medical Center of Hainan Province, Haikou, 570216 China

Correction to: *Scientific Reports* 10.1038/s41598-024-66339-9, published online 07 July 2024

The original version of the Article contained an error in the Author Information section.

“These authors contributed equally: Xianfeng Huang and Da-ya Zhang.”

now reads:

“These authors contributed equally: Xianfeng Huang, Da-ya Zhang and Da Li.”

The original Article has been corrected.

