# Supplementary material for: Human gastric microbiota analysis of refractory H. pylori infection
Source: Sci Rep. 2024 Jul 7;14:15619. doi: 10.1038/s41598-024-66339-9 (PMC11228035; doi:10.1038/s41598-024-66339-9)
Supplement: Supplementary file 1 — Supplementary Figures. [file 41598_2024_66339_MOESM1_ESM.docx]

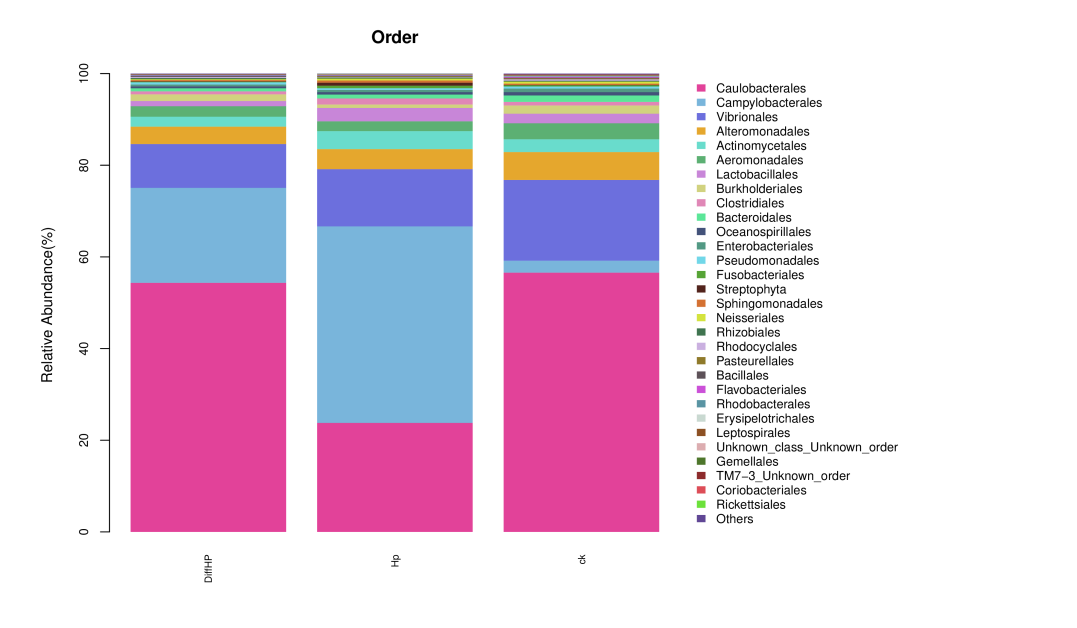


Figure S1 Relative abundance of gastric microbiota Order level among groups.

CK:H.pylori-negative patients;HP: treatment-naïve patients with H. pylori infection;Diff

HP:patients with refractory H. pylori infection.


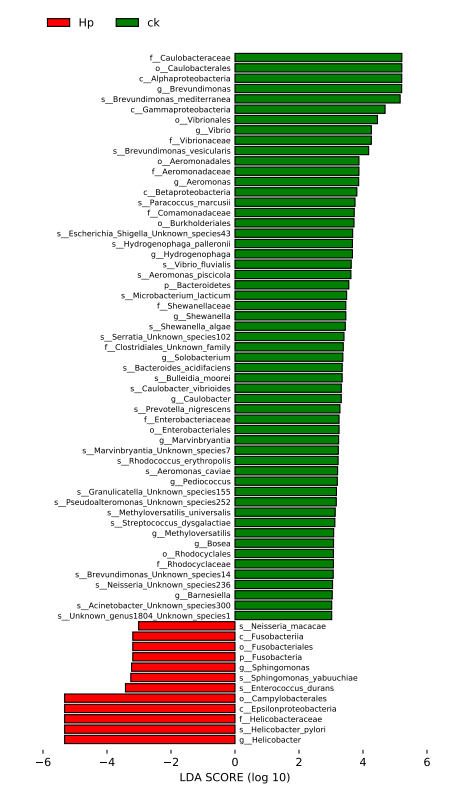


Figure S2A Identification of differential bacteria between Group HP and the Group ck by LEfSe analysis

ck:H.pylori-negative patients;

HP:treatment-naïve patients with H. pylori infection.


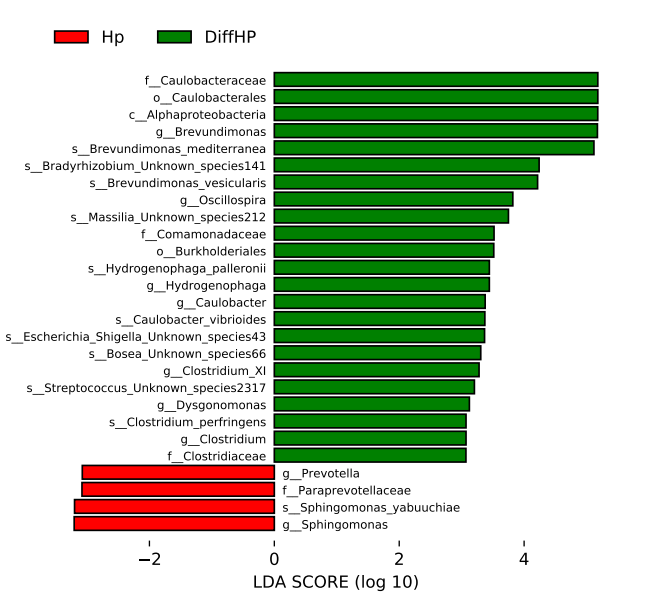


Figure S2B Identification of differential bacteria between Group HP and the Group DiffHP by LEfSe analysis

DiffHP:patients with refractory H. pylori infection;

HP:treatment-naïve patients with H. pylori infection.


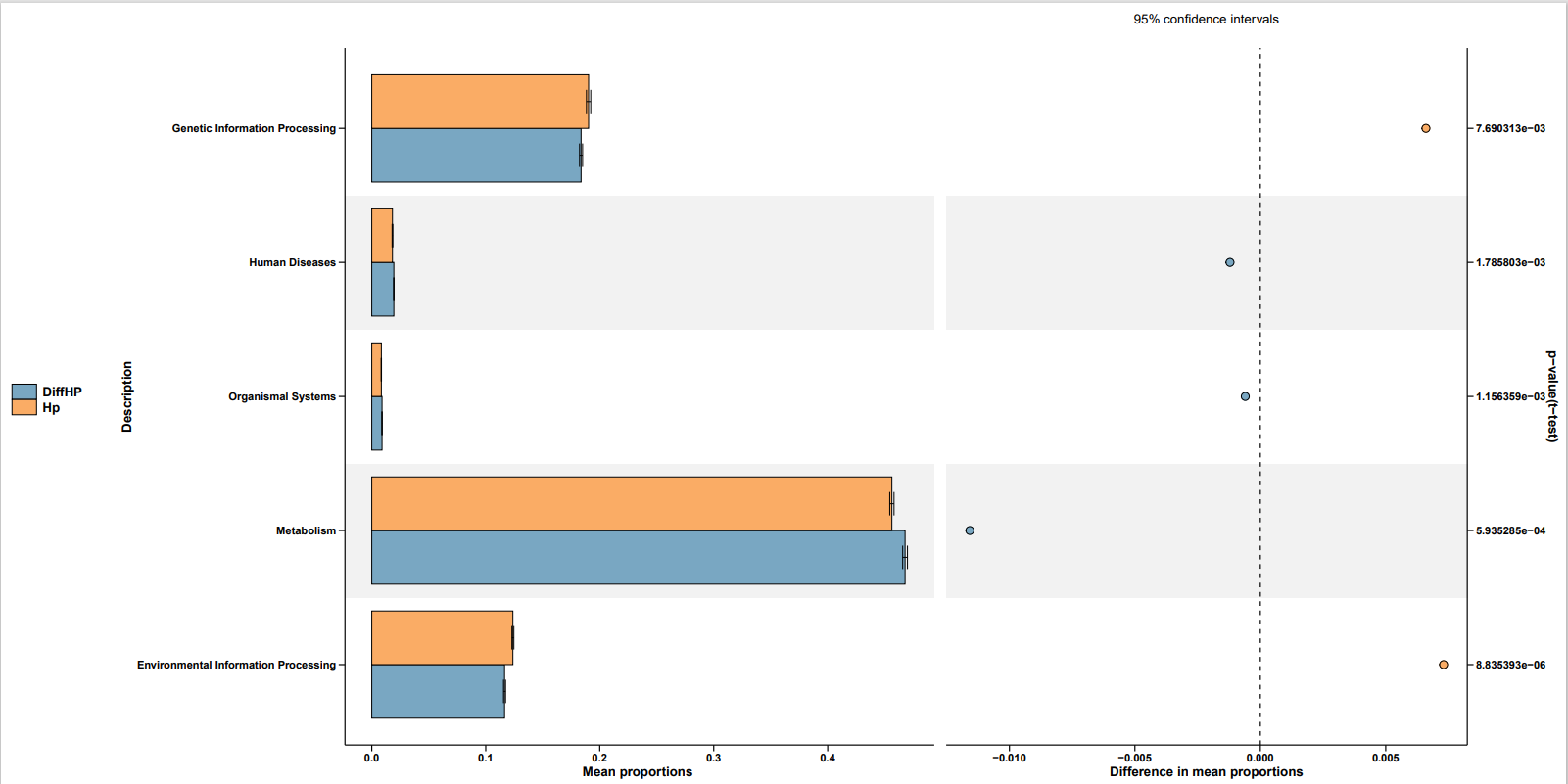


Figure S3A The predicted gastric microbiota function in KEGG pathway at levels 1 between group DiffHP and group Hp.

HP:treatment-naïve patients with H. pylori infection;Diff

HP:patients with refractory H. pylori infection.


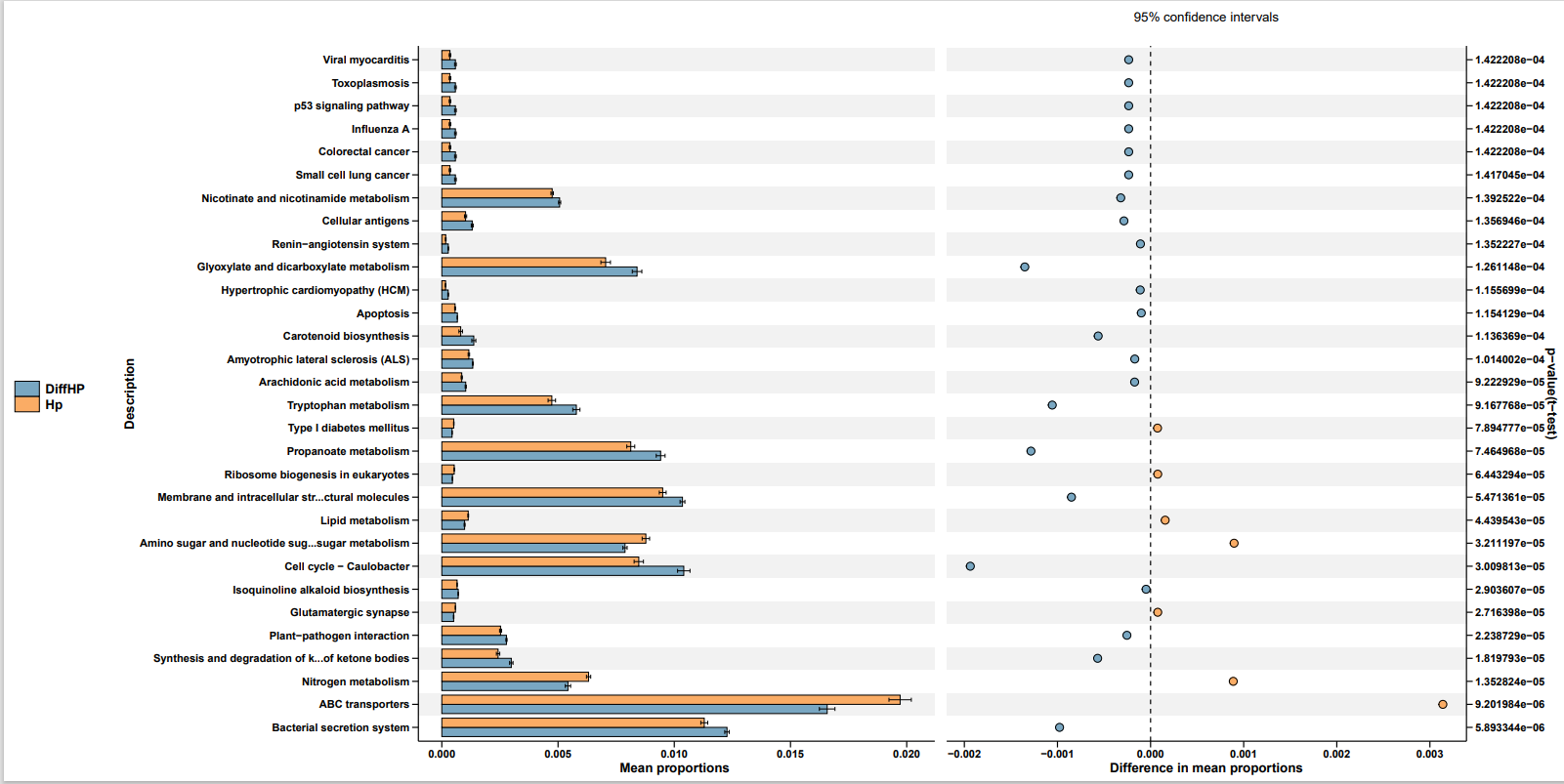


Figure S3B The predicted gastric microbiota function in KEGG pathway at levels 3 between group DiffHP and group Hp.

HP:treatment-naïve patients with H. pylori infection;Diff

HP:patients with refractory H. pylori infection.
